# Effects of dietary *L*-Citrulline or *L*-arginine supplementation on immune function, intestinal morphology and intestinal microbiota in LPS-challenged broilers

**DOI:** 10.1016/j.psj.2026.107307

**Published:** 2026-06-17

**Authors:** Yan Ma, Ming Yang Gao, Shuai Hu Chen, Hong Shen

**Affiliations:** a Shihezi University College of Animal Science, Shihezi 832000, China; b Bingtuan Key Laboratory for Efficient Utilization of Non-Grain Feed Resources, Shihezi 832000, China

**Keywords:** *L*-citrulline, *L*-arginine, Lipopolysaccharide, Immunity, *Akkermansia*

## Abstract

This study aimed to investigate the effects of dietary *L*-citrulline (*L*-Cit) or *L*-arginine (*L*-Arg) supplementation on jejunal mucosal barrier function and inflammatory response in broilers under lipopolysaccharide (LPS) challenge. A total of 384 one-day-old yellow-feathered broilers were randomly divided into 4 groups with 8 replicates per group and 12 birds per replicate. The control group and LPS group were fed a basal diet, while the *L*-Cit group and *L*-Arg group were supplemented with 1% *L*-Cit and 1% *L*-Arg in the basal diet, respectively. The experiment lasted for 27 days. On days 22, 24, and 26 of the experiment, broilers in the control group were intraperitoneally injected with 1 mg/kg body weight of saline, while those in the LPS group, *L*-Cit group, and *L*-Arg group were intraperitoneally injected with 1 mg/kg body weight of LPS. The results showed that no significant effect on growth performance of 21d broilers was observed among all groups (*P* > 0.05). Both the *L*-Cit group and *L*-Arg group significantly or extremely significantly increased the levels of T-AOC, SOD, IgG, and IgM (*P* < 0.05 or *P* < 0.01), and significantly decreased the levels of IL-6 and TNF-α (*P* < 0.05 or *P* < 0.01). Hematoxylin-eosin staining and immunofluorescence analysis revealed that *L*-Cit alleviated intestinal villus atrophy and enhanced intestinal barrier integrity induced by LPS challenge. Notably, both *L*-Cit and *L*-Arg regulated the structure of the intestinal microbial community. *L*-Arg primarily promoted the abundance of beneficial bacteria such as g_*Faecalibacterium* and g_*Barnesiella,* whereas *L*-Cit significantly promoted g_*Akkermansia* to become the dominant genus and exert its function.

## Introduction

Intestinal inflammation significantly impairs the health and performance of livestock and poultry, and is often closely associated with pathogenic microbial infection and intestinal homeostasis imbalance(Ahn et al., 2026). Lipopolysaccharide (LPS), a major component of the Gram-negative bacterial cell wall, induces this response by triggering the release of excessive pro-inflammatory cytokines (e.g., IL-6, TNF-α), leading to oxidative stress and impairment of the intestinal mucosal barrier(Mikołajczyk, et al., 2017; Wang, et al., 2022) . In yellow-feathered broilers, maintaining intestinal structural integrity is crucial for optimal performance and immune function (Bai, et al., 2025). Thus, identifying nutritional interventions that effectively alleviate LPS-induced intestinal inflammation is of great practical significance.

*L*-arginine (*L*-Arg) is a conditionally essential amino acid critical for the antioxidant defense system, immunoglobulin synthesis, and intestinal mucosal repair (Milner, 1985). However, directly supplemented *L*-Arg has limited intestinal absorption and utilization efficiency (Agarwal, et al., 2017). In contrast, *L*-citrulline (*L*-Cit), an efficient precursor for endogenous *L*-Arg synthesis, exhibits extremely high bioavailability (Jiang, et al., 2025). Studies show *L*-Cit effectively regulates intestinal microbiota, enhances immunity, and improves mucosal barriers (Uyanga, et al., 2021).Furthermore, *L*-Cit has been shown to improve feed conversion and ileal- nitrogen digestibility more effectively than *L*-Arg, making it a high-quality alternative source of arginine for broilers (Dao, et al., 2021).

Our previous research confirmed that 1% dietary *L*-Cit supplementation optimally increases intestinal villus height, improving production performance, antioxidant capacity, and immune function. However, under pathological stress like LPS-induced inflammation, it remains unclear whether highly bioavailable *L*-Cit provides equivalent or superior protective effects compared to *L*-Arg, and what their specific mechanistic differences are.

Therefore, this study established an LPS-induced intestinal injury model in yellow-feathered broilers. Based on our prior findings and existing literature(Zhang, et al., 2017), we compared the efficacy of 1% *L*-Cit versus 1% *L*-Arg in alleviating pathological intestinal inflammation. This aims to provide a solid theoretical basis for applying *L*-Cit as an anti-stress functional additive in poultry production.

## Materials and methods

### Experimental materials

*L*-Cit and *L*-Arg were purchased from Shandong Pingju Biotechnology Co., Ltd., in the form of white powder, with a purity of 99.1% and an ash content of 0.05%.

### Animals and experimental design

The experiment was conducted using a completely randomized design. A total of 384 one-day-old broilers were randomly divided into 4 groups: control group (CON), LPS group, LPS+1% *L*-Cit group (Ma, et al., 2025), LPS+1% *L*-Arg group (Zhang, Lv, Li, Guo, Liu and Guo, 2017), with 8 replicates per group and 12 birds per replicate, the experiment lasted for 27 days. On days 22, 24, and 26 of the experiment, broilers in the control group were intraperitoneally injected with 1 mg/kg body weight of saline, while those in the LPS group, *L*-Cit group, and *L*-Arg group were intraperitoneally injected with 1 mg/kg body weight of *E. coli* LPS (Tian, et al., 2026). The LPS from Escherichia coli was purchased from Sigma Chemical Co.(L2880, St. Louis, MO, USA)

During the entire experiment period, all chickens were provided with feed and water ad libitum. The experiment was conducted in a temperature-controlled room at the Animal Experiment Station of Shihezi University (Xinjiang, China). The house temperature was set at 33°C from 1 to 4 days of age, and then reduced by 3°C per week until reaching approximately 24°C. The composition and nutrient levels of the basal diet are presented in Table 1. The basal diet was formulated to meet or exceed the basic nutritional requirements recommended by the NRC (1994). The rationale for employing this standard is threefold: first, yellow-feathered broilers are a slow-growing breed with relatively lower absolute nutritional demands compared to modern fast-growing white-feathered broilers, making this standard physiologically adequate; second, a basal diet without excessive nutrient oversaturation helps prevent masking the specific modulatory effects of the supplemented functional amino acids (*L*-Cit and *L*-Arg); and third, it provides a well-recognized standardized baseline for comparison with previous mechanistic models of intestinal stress and amino acid interventions.

**Table 1:** Ingredient composition and calculated nutrient levels of the basal diet (%, as-fed basis).

Item	1-21 days old	22-27 days old
Ingredients		
Corn	62.00	64.00
Soybean meal	24.00	22.00
Cottonseed meal	6.00	6.00
Wheat bran	3.68	3.68
CaHPO_4_	1.24	1.24
Limestone	1.33	1.33
Salt	0.25	0.25
*L*-Lysine, 99%	1.33	1.33
DL-Methionine,99%	0.29	0.29
*L*-Threonine,99%	0.17	0.17
Premix^1^	1.00	1.00
Nutrient levels^2^		
ME, MJ/kg	13.50	13.00
DM	90.01	90.64
Crude Ash	5.75	6.27
CP	22.26	19.70
EE	3.13	3.67
ADF	4.74	4.81
NDF	11.49	11.41
TP	0.57	0.57
Ca	1.24	1.49
*L*-Arginine	1.21	1.27
Dig^3^ Lysine	0.84	0.80
Dig. Methionine	0.27	0.26
Dig. Threonine	0.58	0.55
Dig. Tryptophan	0.18	0.17

1 Per kg of feed, the premix provides:VitA 180,000 IU; VitD 70,000 IU; VitE 450 IU; VitK 30 mg; VitB 70 mg; niacin 600 mg; calcium pantothenate 260 mg; biotin 1.7 mg; folic acid 17 mg; Fe 10,000 mg; Cu 350 mg; Mn 1500 mg; Zn 2000 mg; Ca 14 mg; P 6 mg; sodium chloride 7 mg; and methionine 3 mg.

2 Metabolic energy is the calculated value, Arginine and the other indicators are measured values,and the other indicators are measured values.

3 Digestible amino acid coefficients for raw ingredients were derived from the Evonik AMINONIR® Advanced calibration database, which is based on near-infrared reflectance spectroscopy (NIRS) analysis standardized with a Foss NIR 6500 instrument (Denmark).

### Sample collection

On day 27, six broilers of moderate body weight (one bird from each replicate) were randomly selected from each treatment. Blood samples were collected from the wing veins and centrifuged at 3,000 rpm for 15 minutes. The supernatant serum was aliquoted and stored at −20°C for serum biochemical indicator analysis. The broilers were euthanized by cervical dislocation, and jejunal tissue samples were collected. A 2-cm section of the mid-jejunum was collected, gently flushed with 4°C phosphate-buffered saline (PBS) to remove contents, and then fixed in 4% paraformaldehyde for morphological analysis. The remaining jejunal segment was opened longitudinally and flushed with ice-cold phosphate-buffered saline to remove contents. The mucosa of each sample and cecal contents were collected using a sterile glass microscope slide, immediately placed in liquid nitrogen, and subsequently stored at −80°C for further analysis.

### Growth performance

BW and feed intake (FI) of broilers were measured on days 1 and 21 of the trial and FCR, ADG, and ADFI were calculated.

### Antioxidant and immune indexes

The plasma levels of immunoglobulin A (IgA), immunoglobulin G (IgG), immunoglobulin M (IgM), interleukin-1β (IL-1β), interleukin-6 (IL-6), and tumor necrosis factor-α (TNF-α) were determined using enzyme-linked immunosorbent assay. The plasma levels of LPS, DAO, malondialdehyde (MDA), superoxide dismutase (SOD), and total Antioxidant Capacity (T-AOC) were determined using colorimetric method. All procedures were performed strictly in accordance with the instructions of the kits (purchased from Beijing Huaying Biotechnology Research Institute).

### Jejunal histomorphology

The jejunal segments were dehydrated, cleaned, and fixed in buffered formalin for 24 hours, then embedded in paraffin and sectioned. Serial sections were cut at a thickness of 5 μm for hematoxylin and eosin staining. Villus width (VW) and villus area (VA) were measured using a biological microscope (Eclipse Ci-L, Nikon Corporation, Japan). Lymphocyte density and Goblet Cell density were calculated using Image-Pro Plus 6.0 analysis software, with millimeters (mm) as the standard unit. Lymphocyte density was calculated as the number of lymphocytes (cells)/ epithelial length (mm), and goblet cell density was calculated as the number of goblet cells (cells) / epithelial length (mm).

### Extraction of RNA and quantitative real-time PCR (qRT-PCR) analysis

A TRIzol kit (TransGen, Beijing, China) was employed to extract jejunal mucosa total RNA and its purity and concentration were determined spectrophotometrically (Thermo Fisher Scientific, Waltham, MA, US). A reverse transcription kit was used to convert total RNA into cDNA, which was analyzed in a 20 µL reaction containing 15 µL of PerfectStart Green qPCR SuperMix, 10 ng of cDNA, and 0.2 µM forward and reverse primers on a Roche LightCycler 96. Thermocycling conditions: initial denaturation at 95°C for 30 s, followed by 50 cycles of denaturation at 95°C for 10 s, annealing at 60°C for 15 s, elongation at 72°C for 10 s. Samples were run in triplicate, and relative expression was determined using the 2^−∆∆Ct^ method. Primers for the broiler genes, TNF-α, IL-1β, IL-6, Occludin, Claudin-1, and ZO-1 were used, with β-actin serving as the reference gene (Table 2).

**Table 2:** Primer sequences of target genes and the reference gene, β-actin.

Gene name	Primer sequence (5′−3′)	GenBank ID	PCR Products (bp)
TNF-α	F: GGCAATGAACCCTCCCCAGTA R: GGTTACAGGAAGGGCAACTCATC	XM_015294121.4	148
IL-1β	F: CAGCCTCAGCGAAGAGACCTT R: ACTGTGGTGTGCTCAGAATCC	XM_015297469.3	294
IL-6	F: CGCCTTTCAGACCTACCT R: GGATTGTGCCCGAACTAA	NM_204628.2	241
occludin	F: ACGGCAGCACCTACCTCAA R: GGGCGAAGAAGGAGATGAG	XM_025144247.2	579
claudin 1	F: TTCATGATGCCTGCTCTTGTG R: CCTGAGCCTTGGTACATTCTTGT	NC_052540.1	2578
MUC2	F: GGGATGTTTATTTGGGCGGC R: TCACCGTGTGTTGTTCCCAT	XM_040673054.2	4767
β-Actin	F: GAGAAATTGTGCGTGACATCA R: CCTGAACCTCTCATTGCCA	NM_204305.1	1287

Abbreviation:TNF-α, tumor necrosis factor alpha; IL-1β, interleukin 1 beta; IL-6,interleukin 6; MUC2,Mucin 2; F, forward primer; R, reverse primer.

### Short-chain fatty acid analysis

The short-chain fatty acids (SCFAs; acetate, propionate, butyrate, valerate) were determined by gas chromatography. After mixing, 0.4 g of cecal contents was added to 1.0 mL of distilled water, mixed thoroughly by pipetting, and centrifuged at high speed. The supernatant was collected for analysis. 4-Methylvaleric acid was used as an internal standard, and the concentrations of volatile fatty acids were measured using a Shimadzu GC2010 gas chromatograph equipped with a Stabilwax column(Li, et al., 2021).

### 16S rRNA sequencing for microbiota analysis

Cecal microbial diversity: Bacterial metagenomic DNA was extracted from cecal contents collected at slaughter using a DNA extraction kit (TIANGEN, Beijing, China). Concentration and purity were assessed using a NanoDrop 2000 microplate reader, and integrity was verified by 1% agarose gel electrophoresis. PCR amplification of the V3-V4 variable region of the 16S rRNA gene was performed using primers 338F (5′-ACTCCTACGGGAGGCAGCAG-3′) and 806R (5′-GGACTACHVGGGTWTCTAAT-3′). The PCR products were recovered, purified using the AxyPrep DNA Gel Extraction Kit (Axygen Biosciences), and quantified using the QuantiFluor™-ST system (Promega). Sequencing was performed on the Illumina MiSeq PE300 platform.

After sequencing, the raw data were subjected to quality control, trimming, denoising, merging, and chimera removal using the QIIME2 pipeline to obtain the final amplicon sequence variants (ASVs). The QIIME2 feature-classifier plugin was used to align representative sequences against the GREENGENES database for taxonomic classification. All downstream statistical analyses and visualizations were performed using the NovoCloud platform (https://magic-plus.novogene.com/#/).

Statistical analysis of microbiota data: Alpha diversity indices were calculated to evaluate species richness and evenness, and differences among groups were strictly analyzed using the non-parametric Kruskal-Wallis sum-rank test. For Beta diversity, Principal Coordinate Analysis (PCoA) based on distance matrices was used to evaluate differences in microbial community structure. The statistical significance of these structural variations among the treatment groups was rigorously validated using PERMANOVA (Adonis function) with 999 permutations. To identify significantly differential bacterial taxa among groups, the Analysis of Composition of Microbiomes (ANCOM) method and Linear discriminant analysis Effect Size (LEfSe) were employed. For LEfSe, a stringent LDA score threshold of >4.0 was applied to identify robust biomarker taxa. Furthermore, to control the false discovery rate during multiple taxonomic comparisons, P-values were adjusted using the Benjamini-Hochberg (FDR) method, with statistical significance defined at an adjusted *P* < 0.05.

### Statistical analysis

The experimental data were initially sorted by Excel 2010, and the ANOVA program of SPSS 26.0 was used for one-way ANOVA analysis. If the differences were significant, Tukey's method was used for multiple comparison,The test results were expressed as mean and standard error (SEM); *P* < 0.05 was the significant difference level, and *P* < 0.01 was the extremely significant difference level. Pearson correlation analysis was used to calculate correlation coefficients, and the heatmap tool in Hiplot Pro (https://hiplot.com.cn/) was used to generate correlation heatmaps. Relative abundances of bacteria and short-chain fatty acids, as well as Spearman correlation coefficients, were analyzed and visualized using GraphPad Prism 10.1.2.

## Results

### Growth performance

As shown in Table 3, supplementation with *L*-Cit and *L*-Arg had no significant effect on initial body weight, final body weight, ADFI, ADG, or F/G of broilers at 21 days of age (*P* > 0.05).

**Table 3:** Effect of dietary supplementation with *L*-Cit and *L*-Arg on growth performance of broilers.

Items	Groups					SEM	*P*-Value
	CON	LPS	*L*-Cit	*L*-Arg			
BW at 1 day of age	30.72	30.99	30.41		30.55	1.508	0.932
BW at 21 day of age	549.50	540.00	586.66		596.66	13.696	0.403
ADFI, g	32.75	31.91	31.65		30.58	2.406	0.505
ADG, g	24.70	24.24	26.48		26.96	3.201	0.397
FCR	1.34	1.35	1.21		1.15	0.202	0.250

Abbreviation: BW: Body Wight, ADFI: Average Daily Feed Intake**,** ADG: Average Daily Gain, FCR: Feed Conversion Ratio.

Note:CON, basal diet; LPS,injected with 1 mg/kg body weight of *E. coli* LPS; *L*-Cit,basal diet supplemented with 1% *L*-Cit; *L*-Arg,basal diet supplemented with 100 mg/kg *L*-Arg.

### Serum immune and antioxidant indexes

As shown in Table 4, in terms of antioxidant indices, *L*-Cit+LPS and *L*-Arg+LPS significantly increased the levels of T-AOC and SOD (*P* < 0.05 or *P* < 0.01); in terms of immune indices, *L*-Cit+LPS and *L*-Arg+LPS extremely significantly increased the levels of IgG and IgM (*P* < 0.01); in terms of inflammatory factors, *L*-Cit+LPS and *L*-Arg+LPS significantly decreased IL-6 and TNF-α (*P* < 0.05 or *P* < 0.01); in terms of intestinal permeability, compared with the CON group, LPS challenge increased the serum levels of DAO and LPS; both *L*-Cit and *L*-Arg supplementation reduced the serum levels of DAO and LPS, but the differences were not significant compared with the LPS group.

**Table 4:** Effects of dietary supplementation with *L*-Cit and *L*-Arg on antioxidant and immune indices of broilers.

Items	Groups					SEM	*P-*Value
	CON	LPS	*L*-Cit+LPS	*L*-Arg+LPS			
T-AOC, U/ml	8.96^a^	5.12^b^	7.48^a^		7.46^a^	0.409	0.012
SOD, U/ml	137.12^Aa^	95.10^Bb^	126.21^Aa^		131.59^Aa^	4.074	<0.001
MDA, nmol/ml	2.74	4.20	3.40		3.09	0.282	0.317
IgA, μg/L	446.97	416.77	483.04		466.72	11.759	0.225
IgG, μg/L	654.43^ABa^	599.74^Bb^	678.32^Aa^		632.40^ABab^	9.205	0.009
IgM, μg/L	272.68 ^ABa^	235.78^Bb^	292.60^Aa^		276.25^ABab^	6.527	0.008
IL-1β, pg/ml	140.10	156.12	121.68		140.66	5.467	0.172
IL-6, pg/ml	159.30^b^	200.18 ^a^	147.36^b^		173.86^ab^	6.989	0.034
TNF-α, pg/ml	92.80^Bb^	117.08^Aa^	86.14^Bb^		94.53^ABb^	3.595	0.006
DAO, ng/mL	1.01	1.07	0.98		1.07	0.029	0.692
LPS, ng/mL	1.42	1.66	1.38		1.48	0.055	0.309

Abbreviation: T-AOC: Total Antioxidant Capacity, SOD: superoxide dismutase, MDA: malondialdehyde,IgA: immunoglobulin A, IgG: immunoglobulin G, IgM: immunoglobulin M, IL-1β: interleukin-1β, IL-6: interleukin-6, TNF-α: tumor necrosis factor-α.

Note: ^a,b^ Values in the same row with no common superscript differ significantly (*P* < 0.05).

^A,B^ Values in the same row with no common superscript differ significantly (*P* < 0.01). CON, basal diet; LPS,injected with 1 mg/kg body weight of *E. coli* LPS; *L*-Cit, basal diet supplemented with 1% *L*-Cit and injected with 1 mg/kg body weight of *E. coli* LPS. *L*-Arg,basal diet supplemented with 1% *L*-Arg and injected with 1 mg/kg body weight of *E. coli* LPS.

### *L*-Cit and *L*-Arg alleviates LPS-induced acute intestinal injury and inflammation

**Fig. 1:**
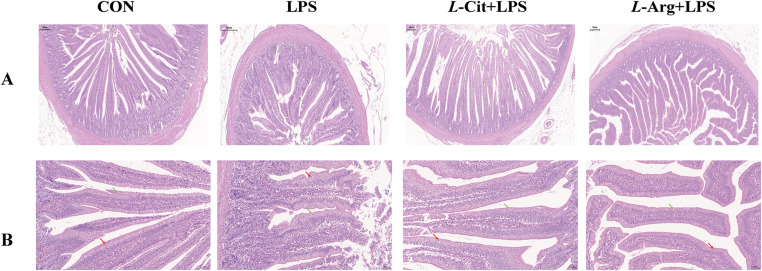
(A) H&E staining of jejunal tissue sections (Bar scale upper 500 μm, lower 80 μm). (B) The number of goblet cells and lymphocytes, with green arrows indicating goblet cells and red arrows indicating lymphocytes.CON, basal diet; LPS,injected with 1 mg/kg body weight of *E. coli* LPS; *L*-Cit, basal diet supplemented with 1% *L*-Cit and injected with 1 mg/kg body weight of *E. coli* LPS. *L*-Arg,basal diet supplemented with 1% *L*-Arg and injected with 1 mg/kg body weight of *E. coli* LPS.

As shown in Table 5, the results of H&E staining and immunofluorescence staining showed that LPS injection alone significantly decreased VH, while supplementation with *L*-Cit significantly alleviated intestinal villus atrophy induced by LPS stress and increased VH (*P* < 0.05), no significant difference was observed between the *L*-Arg group and the LPS group (Fig. 1A). In terms of cell density, compared with the CON group, LPS challenge alone significantly decreased the cell density of lymphocytes and goblet cells in the jejunum; both *L*-Cit and *L*-Arg supplementation increased the cell density of these two cell types, restoring them to levels close to those of the CON group (Fig. 1B).

**Table 5:** Effects of dietary supplementation with *L*-Cit or *L*-Arg on jejunal histomorphology.

Items	Groups				SEM	*P-*Value
	CON	LPS	*L*-Cit+LPS	*L*-Arg+LPS		
Villis height	965.06^aA^	590.76^bB^	902.35^aA^	608.66^bB^	186.134	<0.001
Crypt depth	106.86	106.86	106.86	106.86	30.754	0.633
V/C	9.87	9.87	9.87	9.87	3.095	0.170
Goblet Cell	43.33	38.00	37.00	31.17	9.841	0.201
Lymphocyte	18.33	15.83	19.00	20.17	5.467	0.597

Abbreviation: V/C:Villis height/Crypt depth.

Note:^a,b^ Values in the same row with no common superscript differ significantly (*P* < 0.05).

^A,B^ Values in the same row with no common superscript differ significantly (*P* < 0.01). CON, basal diet; LPS,injected with 1 mg/kg body weight of *E. coli* LPS; *L*-Cit, basal diet supplemented with 1% *L*-Cit and injected with 1mg/kg body weight of *E. coli* LPS. *L*-Arg,basal diet supplemented with 1% *L*-Arg and injected with 1 mg/kg body weight of *E. coli* LPS.

**Fig. 2:**
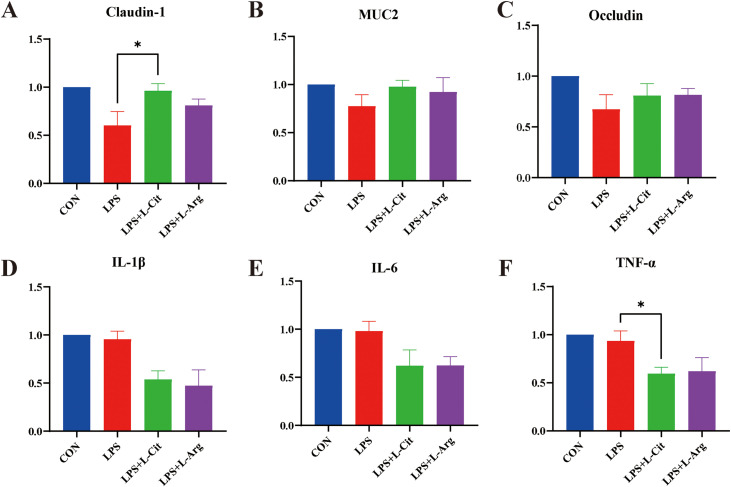
(A) Claudin-1 mRNA expression in jejunal mucosa. (B) MUC2 mRNA expression in jejunal mucosa. (C) Occludin mRNA expression in jejunal mucosa. Inflammatory factors: IL-1β: interleukin-1β (D), IL-6: interleukin-6 (E), TNF-α: tumor necrosis factor-α (F).*P < 0.05, **P < 0.01. CON, basal diet; LPS,injected with 1 mg/kg body weight of *E. coli* LPS; *L*-Cit, basal diet supplemented with 1% *L*-Cit and injected with 1 mg/kg body weight of *E. coli* LPS. *L*-Arg,basal diet supplemented with 1% *L*-Arg and injected with 1 mg/kg body weight of *E. coli* LPS.

Regarding the mRNA expression of mucosal tight junction proteins, compared with the CON group, LPS challenge decreased the expression of Claudin-1, whereas *L*-Cit supplementation significantly increased the mRNA expression of Claudin-1 in the jejunal mucosa (*P* < 0.05), and no significant difference was observed between the *L*-Cit group and the CON group (Fig. 2A). Regarding the mRNA expression of inflammatory cytokines, compared with the CON group, LPS challenge increased the levels of TNF-α, IL-6, and IL-1β in the jejunal tissue; *L*-Cit supplementation significantly reduced the level of TNF-α (*P* < 0.05) (Fig. 2F).

### Short-chain fatty acids

As shown in Table 6, there were no significant effects among groups on Acetate, Propionate, Butyrate, Acetate:Propionate, or Total SCFAs (*P* > 0.05).

**Table 6:** Effects of dietary supplementation with *L*-Cit and *L*-Arg on antioxidant and immune indices of broilers.

Items	Groups					SEM	*P-*Value
	CON	LPS	*L*-Cit+LPS	*L*-Arg+LPS			
Acetate	14.17	13.52	14.00		14.42	0.714	0.063
Propionate	1.00	0.81	1.05		1.08	0.111	0.622
Butyrate	1.16	0.95	1.01		1.28	0.122	0.822
Acetate:Propionate	14.22	23.73	15.80		24.57	2.620	0.396
Total SCFAs	16.42	15.29	16.07		16.95	1.173	0.057

Note:CON, basal diet; LPS,injected with 1 mg/kg body weight of *E. coli* LPS; *L*-Cit, basal diet supplemented with 1% *L*-Cit and injected with 1 mg/kg body weight of *E. coli* LPS. *L*-Arg,basal diet supplemented with 1% *L*-Arg and injected with 1 mg/kg body weight of *E. coli* LPS.

### Microbial composition

As shown in Table 7, the Dominance index in the LPS group was significantly higher than that in the CON and *L*-Arg+LPS groups (*P* < 0.05), and the Simpson index in the *L*-Arg+LPS group was significantly higher than that in the CON and LPS groups (*P* < 0.05).

**Table 7:** Effect of dietary level of *L*-Cit or *L*-Arg on microbial diversity in cecum of broilers.

Items	Groups				SEM	*P-*Value
	CON	LPS	*L*-Cit+LPS	*L*-Arg+LPS		
Chao1	397.08	374.34	337.58	444.74	16.899	0.149
Observed_features	390.00	364.50	330.50	434.17	16.539	0.152
Shannon	5.97	5.68	5.77	6.28	0.103	0.168
Pielou_e	0.69	0.67	0.69	0.72	0.008	0.185
Goods coverage	1.00	1.00	1.00	1.00	0.000	0.235
Dominance	0.04^ab^	0.05^a^	0.04^ab^	0.03^b^	0.004	0.044
Simpson	0.96^ab^	0.95^b^	0.96^ab^	0.97^a^	0.004	0.044

Note: ^a,b^ Values in the same row with no common superscript differ significantly (*P* < 0.05). CON, basal diet; LPS,injected with 1 mg/kg body weight of *E. coli* LPS; *L*-Cit, basal diet supplemented with 1% *L*-Cit and injected with 1 mg/kg body weight of *E. coli* LPS. *L*-Arg,basal diet supplemented with 1% *L*-Arg and injected with 1 mg/kg body weight of *E. coli* LPS.

As shown in Table 8, Bacteroidota and Firmicutes were the dominant phyla at the phylum level, and the relative abundance of Desulfobacterota in the CON group was extremely significantly higher than that in the other experimental groups (*P* < 0.05).

**Table 8:** Effects of dietary supplementation with *L*-Cit and *L*-Arg on cecal bacterial phylum level of broilers.

Items	Groups				SEM	*P-*Value
	CON	LPS	*L*-Cit+LPS	*L*-Arg+LPS		
Bacteroidota	66.24	62.77	66.55	62.72	1.681	0.782
Firmicutes	25.33	31.15	24.68	27.02	0.981	0.074
F/B	0.39	0.50	0.39	0.46	0.026	0.337
Campylobacterota	1.22	0.35	1.52	3.87	0.694	0.333
Deferribacterota	0.41	0.70	0.84	1.84	0.389	0.622
Verrucomicrobiota	0.99	1.04	3.66	1.44	0.416	0.060
Desulfobacterota	2.29^Aa^	0.86^ABb^	0.49^Bb^	0.74^Bb^	0.200	0.001
Synergistota	2.16	1.41	0.49	1.10	0.235	0.078
Proteobacteria	0.90	1.27	1.70	0.98	0.168	0.336
Cyanobacteria	0.31	0.10	0.02	0.05	0.049	0.149

Abbreviation: F/B:Firmicutes/Bacteroidota.

Note: ^a,b^ Values in the same row with no common superscript differ significantly (*P* < 0.05).

^A,B^ Values in the same row with no common superscript differ significantly (*P* < 0.01). CON, basal diet; LPS,injected with 1 mg/kg body weight of *E. coli* LPS; *L*-Cit, basal diet supplemented with 1% *L*-Cit and injected with 1 mg/kg body weight of *E. coli* LPS. *L*-Arg,basal diet supplemented with 1% *L*-Arg and injected with 1 mg/kg body weight of *E. coli* LPS.

As shown in Table 9, the relative abundance of Bacteroides in the *L*-Arg+LPS group was significantly lower than that in the other groups (*P* < 0.05), while the relative abundances of Faecalibacterium and Barnesiella were significantly higher than those in the other groups (*P* < 0.05); the relative abundance of Akkermansia in the *L*-Cit+LPS group was significantly higher than that in the other groups (*P* < 0.05).

**Table 9:** Effects of dietary supplementation with *L*-Cit and *L*-Arg cecal bacterial genus level of broilers.

Items	Groups				SEM	*P-*Value
	CON	LPS	*L*-Cit+LPS	*L*-Arg+LPS		
*Bacteroides*	50.49^Aa^	49.76^ABa^	43.46^ABab^	33.13^Bb^	2.141	0.006
*Alistipes*	4.68	4.34	3.24	1.48	0.482	0.071
*Prevotellaceae_NK3B31_group*	5.77	2.86	4.18	4.31	0.571	0.373
*Ligilactobacillus*	0.61	3.56	3.88	1.29	0.664	0.214
*Faecalibacterium*	2.55^ab^	0.73^b^	1.03^b^	4.76^a^	0.513	0.010
*Barnesiella*	0.11^Bb^	0.62^Bb^	1.26^Bb^	6.17^Aa^	0.632	<0.001
*Helicobacter*	0.53	0.22	1.33	2.77	0.489	0.262
*Mucispirillum*	0.41	0.70	0.84	1.84	0.389	0.622
*Phascolarctobacterium*	4.23	4.30	2.54	1.93	0.420	0.097
*Akkermansia*	0.20^b^	0.74^b^	2.55^a^	0.57^b^	0.335	0.047

Note: ^a,b^ Values in the same row with no common superscript differ significantly (*P* < 0.05).

^A,B^ Values in the same row with no common superscript differ significantly (*P* < 0.01). CON, basal diet; LPS,injected with 1 mg/kg body weight of *E. coli* LPS; *L*-Cit, basal diet supplemented with 1% *L*-Cit and injected with 1 mg/kg body weight of *E. coli* LPS. *L*-Arg,basal diet supplemented with 1% *L*-Arg and injected with 1 mg/kg body weight of *E. coli* LPS.

**Fig. 3:**
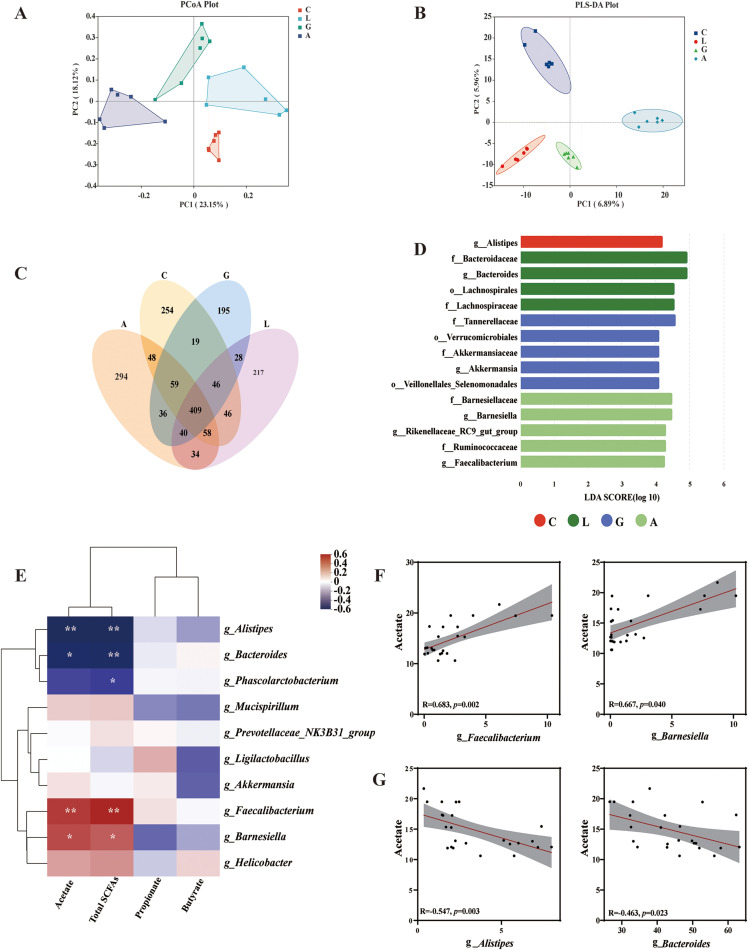
Effects of dietary supplementation with *L*-citrulline and *L*-arginine on cecal gut microbiota composition of yellow-feathered broilers. (A) PCoA analysis. (B) PLS-DA analysis. (C) Venn diagram of cecal microbiota in yellow-feathered broilers. (D) LEfSe analysis. (E) Heatmap of correlation between cecal bacteria at genus level and short-chain fatty acids, *P < 0.05, **P < 0.01. (F-G) Results of correlation analysis between acetate and g_*Faecalibacterium*, g_*Barnesiella*, g_*Alistipes,* g_*Bacteroides*.CON, basal diet; LPS,injected with 1 mg/kg body weight of *E. coli* LPS; *L*-Cit, basal diet supplemented with 1% *L*-Cit and injected with 1 mg/kg body weight of *E. coli* LPS. *L*-Arg,basal diet supplemented with 1% *L*-Arg and injected with 1 mg/kg body weight of *E. coli* LPS.

As shown in Fig. 3, in the PCoA analysis (Fig. 3A), PC1 was 23.15% and PC2 was 18.12%, with a cumulative contribution rate of 41.27%, indicating that the reduced-dimensional data explained 41.27% of the original data. In addition, the analysis of the reduced-dimensional data showed clear separation among groups, indicating significant differences in gut microbiota among the experimental groups. Similarly, the results presented in the PLS-DA plot (Fig. 3B) were consistent with those of the PCoA analysis. OTU analysis, a Venn diagram of each sample was obtained according to different groups (Fig. 3C): there were 409 common OTUs among the four groups, with 254 unique OTUs in the CON group, 217 unique OTUs in the LPS group, 195 unique OTUs in the *L*-Cit+LPS group, and 294 unique OTUs in the *L*-Arg+LPS group. Linear discriminant analysis effect size (LEfSe) showed (Fig. 3D) that, with an LDA score of 4, the CON group had one potential gut microbiota biomarker (g_*Alistipes*); the LPS group had four potential gut microbiota biomarkers (f_Bacteroidaceae, g_*Bacteroides*, o_Lachnospirales, f_Lachnospiraceae); the *L*-Cit+LPS group had five potential gut microbiota biomarkers (f_Tannerellaceae, o_Verrucomicrobiales, f_Akkermansia, g_Akkermansia); and the L-Arg+LPS group had five potential gut microbiota biomarkers (f_Barnesiellaceae, g_*Barnesiella*, g_*Rikenellaceae_RC9_gut_group*, f_Ruminococcaceae, g_*Faecalibacterium*). To further determine the interaction between gut microbiota and short-chain fatty acids, we performed Pearson correlation coefficient analysis (Fig. 3E). At the genus level, Acetate was significantly negatively correlated with g_*Faecalibacterium* and g_*Barnesiella* (*P* = 0.002 and *P* = 0.040), and significantly positively correlated with g_*Alistipes* and g_*Bacteroides* (*P* = 0.003 and *P* = 0.023).

## Discussion

Adding appropriate amino acids to the animal diet can increase feed intake and enhance the utilization of nutrients(Li and Wu, 2020). In the present study, dietary supplementation with 1% *L*-Cit or 1% *L*-Arg had no significant effect on 21-day body weight in yellow-feathered broilers, which is consistent with previous reports Specifically, dietary 1.2% *L*-arginine has been shown to positively regulate hepatic and blood metabolite profiles without affecting 21-day body weight(Brugaletta, et al., 2023). Likewise, supplementation with arginine (1.15%, 1.25%) or citrulline (0.38%, 0.58%) did not significantly affect 21-day body weight but significantly increased 32-day body weight(Lee, et al., 2026).The period before 21 days of age is a critical stage for the development of digestive organs and the establishment of the immune system in broilers. As important functional amino acids, *L*-Cit and *L*-Arg participate in pathways such as nitric oxide synthesis and polyamine metabolism in vivo(Rashid, et al., 2020). Exogenous amino acids may preferentially act on aspects such as promoting intestinal mucosal development and enhancing early stress resistance(Zhang, et al., 2019). Therefore, although no significant change in body weight was observed during the early feeding period, *L*-Cit and *L*-Arg may have positive effects on improving intestinal development and stress resistance.

LPS is commonly used as a stimulus to induce oxidative stress and immune stress in animals, primarily by disrupting the original metabolic balance of the body, thereby causing tissue damage(Li, et al., 2018). The results of this study indicated that dietary supplementation with *L*-Cit and *L*-Arg significantly improved antioxidant and immune performance and reduced the levels of inflammatory factors, indicating that both effectively alleviated the negative effects induced by LPS. The specific potential mechanisms are mainly the following two aspects: in terms of antioxidant capacity, *L*-Cit itself acts as an effective hydroxyl radical scavenger, directly eliminating free radicals in the body.(Uyanga, et al., 2020);Meanwhile, after being metabolized in the circulation and transported to the kidney, approximately 75% of *L*-Cit is efficiently converted to *L*-Arg via the urea cycle through the action of argininosuccinate synthase and lyase. This conversion significantly increases the levels of arginine and nitric oxide in the body (Lan, et al., 2020a). As a result, nitric oxide produced from *L*-Arg as a key precursor can further activate the gene expression of downstream antioxidant enzyme systems, endowing both substances with strong antioxidant capacity (Fathima, et al., 2023). In terms of anti-inflammatory and immune regulation, LPS typically promotes TNF-α to activate the nuclear factor-κB (NF-κB) signaling pathway (Lan, et al., 2020b), stimulating monocytes/macrophages to produce large amounts of inflammatory factors such as IL-1, IL-6, and IL-8; Furthermore, nitric oxide continuously produced in the body via the “Cit-NO-Arg cycle” pathway (Imbard, et al., 2023), can effectively regulate the activity of T cells, macrophages, and NK cells, thereby suppressing excessive immune responses and inflammatory reactions (Uyanga, et al., 2023).In summary, *L*-Cit and *L*-Arg reversed the damage caused by LPS by strengthening the antioxidant defense and attenuating the inflammatory signaling pathway.

*L*-Arg has been shown to have antioxidant, anti-inflammatory, and mucosal protective potential (Wu, et al., 2020). However, the mechanism of action of its precursor *L*-Cit in alleviating intestinal damage has not been fully elucidated. This study showed that *L*-Cit significantly alleviated LPS-induced mucosal damage by ameliorating intestinal villus atrophy and increasing the number of lymphocytes and goblet cells. The intestinal mucus layer and epithelial cells form a critical physical barrier that defends against luminal pathogens and harmful substances and maintains intestinal homeostasis, while LPS disrupts this barrier, leading to downregulation of tight junction protein expression and increased intestinal permeability (Jiang, et al., 2021). *L-*Cit reduced the mRNA expression level of TNF-α, which likely contributed to the maintenance of epithelial integrity. These results indicate that *L*-Cit effectively prevents early mucosal damage by maintaining intestinal barrier integrity, immune homeostasis, and oxidative balance.

Alpha diversity analysis is mainly used to determine the microbial richness and microbial diversity of samples, as well as the coverage information of sample microorganisms under the sequencing conditions (Zhang, et al., 2024). The Dominance index is a key indicator reflecting the heterogeneity of the microbial community and the degree of aggregation of core microbiota (Zhang, et al., 2025). In this study, the Dominance index in the LPS group was significantly increased, indicating that LPS induction led to a decrease in the diversity of the intestinal microbiota in broilers. From the perspective of genus level, the relative abundance of Bacteroides increased significantly, and Bacteroides, as a Gram-negative bacterium, has strong tolerance to the inflammatory microenvironment and oxidative stress (Zünd, et al., 2025). LPS induction disrupted the homeostasis of the original gut microbiota, resulting in a decrease in the relative abundance of a large number of anaerobic commensal bacteria (Zheng, et al., 2020). However, Bacteroides can undergo competitive over-enrichment by virtue of its powerful carbohydrate-active enzyme system. It is precisely this significant increase in the relative abundance of a single or a few dominant genera that upregulates the Dominance index. The Simpson index is a key indicator reflecting species richness and evenness in a microbial community (Gao, et al., 2025). The Simpson index is a key indicator reflecting species richness and evenness in a microbial community. In this study the Simpson index was significantly increased after *L*-Arg treatment, indicating that *L*-Arg as a nitrogen-rich functional amino acid may not only be absorbed by the host in the digestive tract but also effectively ameliorate gut microbiota disorder under LPS stress.

Relative abundance analysis at the phylum level revealed a significant decrease in Desulfobacterota within the experimental groups, suggesting that LPS-induced acute systemic stress may impair the mucus secretion function of intestinal goblet cells during the acute phase. As a result, Desulfobacterota experienced a significant decrease in relative abundance due to the lack of corresponding substrate supply (Han, et al., 2023; Ungerfeld, et al., 2024). At the genus level, *L*-Cit and *L*-Arg exhibited different regulatory pathways for gut microbiota: *L*-Cit mainly enriched *Akkermansia*, a genus known to enhance the physical barrier of the intestinal mucosa, while *L*-Arg suppressed the abundance of harmful bacteria and increased the abundance of beneficial bacteria. *L*-Arg increased the relative abundance of *Faecalibacterium* and *Barnesiella*, indicating that arginine, as a selective nitrogen source, increased the abundance of beneficial bacteria such as *Faecalibacterium* and *Barnesiella*. Notably, although the structure of the microbiota changed significantly, the concentrations of acetate and butyrate in the cecum did not change significantly. Further correlation analysis revealed that acetate was significantly positively correlated with *Bacteroides* and *Alistipes*, but significantly negatively correlated with *Faecalibacterium* and *Barnesiella*, which were enriched by *L*-Arg. This may be because the regulatory effect of *L*-Arg on gut microbiota is primarily achieved by providing a nitrogen source to directly shape microbiota composition, while short-chain fatty acids, as metabolic end products, maintain homeostatic balance at the overall level (Kumar, et al., 2025). In contrast, *L*-Cit significantly increased the relative abundance of *Akkermansia*, which was further verified by LEfSe analysis. *Akkermansia*, as a mucin-degrading specialist, can stimulate the repair of the intestinal mucosa(Tiffany, et al., 2026) and upregulate the expression of tight junction proteins(Wade, et al., 2023), which is consistent with the upregulated expression of Claudin-1 mRNA in the ileal mucosa of the *L*-Cit group.

## Conclusion

**Fig. 4:**
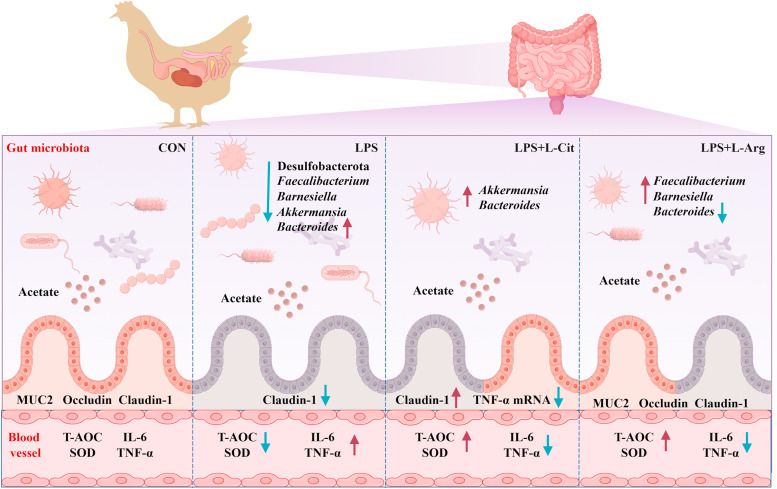
The mechanism by which *L*-Cit or *L-*Arg resist inflammation induced by lipopolysaccharide. CON, basal diet; LPS,injected with 1 mg/kg body weight of *E. coli* LPS; *L*-Cit, basal diet supplemented with 1% *L*-Cit and injected with 1 mg/kg body weight of *E. coli* LPS. *L*-Arg,basal diet supplemented with 1% *L*-Arg and injected with 1 mg/kg body weight of *E. coli* LPS.This figure was drawn using Figdraw and has received a commercial use authorization. Export code: eq=Ar0bc85.

Dietary supplementation with 1% *L*-Cit or *L*-Arg effectively and comparably enhanced the antioxidant capacity and immune function of yellow-feathered broilers under LPS challenge. While both amino acids provided robust systemic protection, they exerted distinct effects on the intestinal mucosa. *L*-Cit showed a specific advantage in alleviating LPS-induced intestinal villus atrophy and maintaining the physical integrity of the mucosal barrier. Furthermore, they differentially modulated the gut microbiota: *L*-Arg increased the relative abundance of Faecalibacterium, whereas *L*-Cit specifically promoted Akkermansia. These findings suggest that while both are effective nutritional interventions for stressed poultry, *L*-Cit may offer targeted benefits specifically for preserving intestinal morphological structure (Fig. 4).

## Ethics statement

The study was approved by the Animal Experimentation Ethics Committee of the college of Animal Science and Technology, Shihezi University. Broilers were housed and handled according to committee guidelines and euthanized humanely. All actions were governed by the principles of minimizing animal suffering (Approval #A2025-1285).

## Ethics approval and consent to participate

The animal study protocol was approved by the Ethics Committee of college of Shihezi University College of Animal Science (NO. A2025-1285).

## Financial support statement

No financial support.

## Data and model availability statement

None of the data were deposited in an official repository. The data is available from the authors upon reasonable request.

## Author contribution

**Yan Ma:** Writing-review & editing, Writing-original daft, Data curation, Conceptualization. **Ming Yang Gao:** Data curation, Conceptualization. **Shuai Hu Chen:** Resources, Methodology, Investigation. **Hong Shen:** Writing-original draft, Validation, Investigation.

## Disclosures

The authors declare no competing financial interests or personal relationships that could have influenced the work reported in this paper.
